# ZFP36L2 Role in Thyroid Functionality

**DOI:** 10.3390/ijms22179379

**Published:** 2021-08-29

**Authors:** Francesco Albano, Valeria Tucci, Perry J. Blackshear, Carla Reale, Luca Roberto, Filomena Russo, Pina Marotta, Immacolata Porreca, Marco Colella, Massimo Mallardo, Mario de Felice, Concetta Ambrosino

**Affiliations:** 1 IEOS-CNR, 80131 Naples, Italy; francesco.albano@biogem.it; 2 Biogem, Istituto di Biologia e Genetica Molecolare, 83031 Ariano Irpino, Italy; valeria.tucci@unicampania.it (V.T.); carla.reale@biogem.it (C.R.); luca.roberto@biogem.it (L.R.); filomena.russo@biogem.it (F.R.); immacolataporreca@gmail.com (I.P.); marco.colella@unisannio.it (M.C.); 3 Department of Precision Medicine, University of Campania “Luigi Vanvitelli”, 80138 Naples, Italy; 4 Signal Transduction Laboratory, National Institute of Environmental Health Sciences, Research Triangle Park, Durham, NC 27709, USA; black009@niehs.nih.gov; 5 Departments of Medicine and Biochemistry, Duke University Medical Center, Durham, NC 27710, USA; 6 Stazione Zoologica Anton Dohrn, 80121 Napoli, Italy; pinamarotta82@gmail.com; 7 Department of Science and Technology, University of Sannio, 82100 Benevento, Italy; 8 Department of Molecular Medicine and Medical Biotechnologies, University of Naples “Federico II”, 80131 Naples, Italy; massimo.mallardo@unina.it

**Keywords:** hypothyroidism, RNA stability, *Zfp36l2* KO, *Nis*, *Notch1*, apoptosis

## Abstract

Thyroid hormone levels are usually genetically determined. Thyrocytes produce a unique set of enzymes that are dedicated to thyroid hormone synthesis. While thyroid transcriptional regulation is well-characterized, post-transcriptional mechanisms have been less investigated. Here, we describe the involvement of ZFP36L2, a protein that stimulates degradation of target mRNAs, in thyroid development and function, by in vivo and in vitro gene targeting in thyrocytes. Thyroid-specific *Zfp36l2*^-/-^ females were hypothyroid, with reduced levels of circulating free Thyroxine (cfT4) and Triiodothyronine (cfT3). Their hypothyroidism was due to dyshormonogenesis, already evident one week after weaning, while thyroid development appeared normal. We observed decreases in several thyroid-specific transcripts and proteins, such as *Nis* and its transcriptional regulators (*Pax8* and *Nkx2*.1), and increased apoptosis in *Zfp36l2*^-/-^ thyroids. *Nis*, *Pax8,* and *Nkx2*.1 mRNAs were also reduced in *Zfp36l2* knock-out thyrocytes in vitro (L2KO), in which we confirmed the increased apoptosis. Finally, in L2KO cells, we showed an altered response to TSH stimulation regarding both thyroid-specific gene expression and cell proliferation and survival. This result was supported by increases in P21/WAF1 and p-P38MAPK levels. Mechanistically, we confirmed *Notch1* as a target of ZFP36L2 in the thyroid since its levels were increased in both in vitro and in vivo models. In both models, the levels of *Id4* mRNA, a potential inhibitor of *Pax8* activity, were increased. Overall, the data indicate that the regulation of mRNA stability by ZFP36L2 is a mechanism that controls the function and survival of thyrocytes.

## 1. Introduction

Congenital hypothyroidism (CH) is a state of inadequate thyroid hormone production at birth. It may involve both thyroid dysgenesis or dyshormonogenesis and defects of thyroid development/position of thyroid hormone (TH) biosynthesis [1]. Different gene mutations can cause CH. Thyroid dysgenesis has been primarily associated with mutations in genes such as those encoding the TSH receptor (*Tshr*) [2], Forkhead box protein E1 (*Foxe1*) [3], and the Paired box protein Pax-8 (*Pax8*) [4,5]. Dyshormonogenesis has been associated with mutations in Sodium/iodide cotransporters (*Slc5a5* or *Nis*), Thyroid peroxidase (*Tpo*), Dual oxidase 2 (*Duox2*), Dual oxidase maturation factor 2 (*Duoxa2*), etc. [6]. Although *Pax8*^-/-^, *Nkx2*.1^-/-^, and *Foxe1*^-/-^ mice reveal the importance of these genes in thyroid development [7], few germline mutations have been detected in the corresponding genes in human patients [8,9,10,11,12,13]. Papers published in the past ten years on this issue suggest that mechanisms leading to congenital thyroid dysfunction are heterogeneous [14] and involve several signaling pathways, including *Notch1* [15].

The transcriptional mechanisms controlling thyroid development and function, including epigenetic aspects, have been deeply investigated [16]. Although the regulation of mRNA stability is also an important mechanism regulating differential gene expression, particularly in developmental processes [17], its role in congenital hypothyroidism has been neglected. Mechanistically, it can be controlled by the binding of RNA-binding proteins (RBP) to sequence elements such as Adenylate-Uridylate- Rich Elements (ARE), present in the 3′UTR of regulated transcripts [18]. The roles of two RBPs, AUF1 and ELAV-like protein 1 (HUR), have been investigated in thyroid carcinogenesis [19] but not in CH. Recently, one or more AREs have been found in silico in the 3′UTR region of several important thyroid transcripts, including *Nis* and *Tshr* mRNAs and their transcriptional regulators, *Nkx2.1* and *Pax8* [19]. Furthermore, regulation of *Nis* mRNA stability has been reported in response to iodine, both in vivo and in vitro [20,21].

The tristetraprolin (TTP) family members, ZFP36, ZFP36L1and ZFP36L2 are CCCH tandem zinc finger (TZF) proteins that promote the decay of their mRNA targets [22]. Some rodents possess an additional member, ZFP36L3, specifically expressed in the placenta and yolk sac [23]. TTP family proteins were found to act as regulators of apoptosis and are involved in cell differentiation [24,25,26]. Gene targeting studies in mice defined distinct roles for the ZFP36 family members, for example, revealing that ZFP36L2 is a key regulator of hematopoiesis [27,28,29]. It also plays a role in female fertility, and its inactivation has been associated with the impairment of lipid metabolism [30,31]. To date, its involvement in thyroid development and function has not been reported. We have previously observed that *Zfp36l2* mRNA was enriched in mouse thyroid buds (E10.5) [32] and decreased in models of thyroid carcinogenesis [33]. Furthermore, *Zfp36l2* expression was reduced in thyrocytes exposed to environmental stressors [34]. 

Here, we report that *Zfp36l2* gene inactivation in vivo and in vitro affects thyroid function and thyrocyte survival by mechanisms involving *Notch1* signaling.

## 2. Results

### 2.1. In Vivo Deletion of Zfp36l2 Impairs Thyroid Function

**Figure 1:**
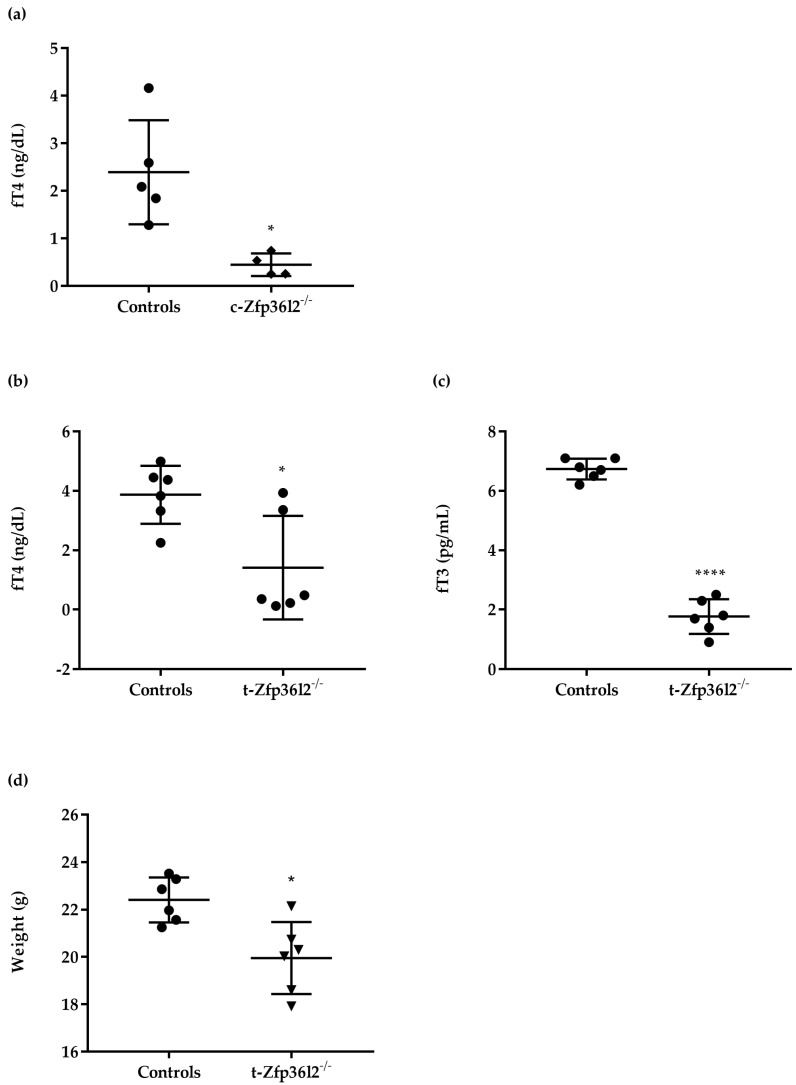
Phenotype of constitutive and thyroid-specific *Zfp36l2*^-/-^ mice. The levels of cfT4 were determined by ELISA in blood collected from the controls (*n* = 5) and conventional *Zfp36l2*^-/-^ (*Zfp36l2*^-/-^ *n* = 4) female mice at PND 21 (**a**). The levels of cfT4 (**b**) and cfT3 (**c**) were determined by ELISA in blood collected from controls (*n* = 6) and t−*Zfp36l2*^-/-^ (*n* = 6) female mice at the PND 90. Body weight evaluation of the t−*Zfp36l2*^-/-^ mice measurements were collected at PND 90 (**d**). Data are reported as mean ± standard deviation * *p* < 0.05; **** *p* < 0.0001.

We started the analysis of the role of ZFP36L2 in thyroid development and function by using a complete knockout mouse model of *Zfp36l2* [29]. As previously reported, complete *Zfp36l2*^-/-^ mice died within approximately three weeks of birth before weaning. ELISA measurement of circulating thyroid hormones in serum samples did not show any major differences in circulating free T4 (cfT4) levels between *Zfp36l2*^-/-^ and control mice at postnatal day seven (PND 7, data not shown). cfT4 levels were reduced in *Zfp36l2*^-/-^ mice at PND 21 (Figure 1a), possibly because the THs supplied by the mother, through lactation, were no longer sufficient. Since no males were available, we evaluated only females (*n* = 4 *Zfp36l2*^-/-^ and *n* = 5 control mice). Thyroids appeared to develop normally and were not ectopic in the *Zfp36l2*^-/-^ mice at PND 21. H&E staining of the sections of the thyroid gland showed a trend towards a decrease in the numbers of follicles together with an increased presence of fat in the thyroid of the *Zfp36l2*^-/-^ mice (Figure S1a, a^1^) vs. controls (Figure S1b, b^1^).

**Figure 2:**
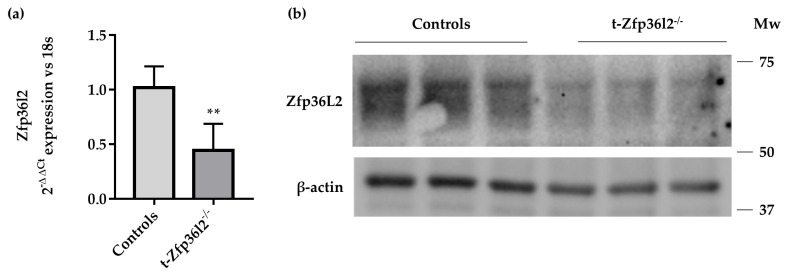
Molecular validation of the t−*Zfp36l2*^-/-^ mice. Analysis of (**a**) *Zfp36l2* mRNA by RTqPCR and (**b**) protein by Western blotting was conducted on extracts from thyroid samples of the control (*n* = 3) and t−*Zfp36l2^-/-^* (*n* = 3) mice. RTqPCR data are reported as mean ± standard deviation of ∆∆Ct values (FC values of gene normalized vs. FC values of 18SrRNA to obtain ∆Ct values of samples; ∆Ct values of KO samples are then normalized vs. ∆Ct of Control samples to obtain ∆∆Ct values) in KO (*n* = 3) and control mice (*n* = 3); ** *p* < 0.01.

To investigate the role of ZFP36L2 in thyroid function at later life stages, we developed mice on a C57/BL6 strain background with thyroid-specific inactivation of *Zfp36l2* (t-*Zfp36l2*^-/-^ from now on). These mice were obtained by crossing the *Zfp36l2*^flox/flox^ mice [35] with the mice expressing a *Pax8-Cre* allele, driving gene inactivation mainly in the thyroid and kidney [36]. Mice were genotyped as described in M&M. As shown by semi-quantitative reverse transcription real-time PCR (RTqPCR) and Western blotting, the deletion of the gene in the whole thyroid of the t-*Zfp36l2*^-/-^ females at PND 90 was not complete, possibly reflecting continued expression in non-thyrocytes. Nevertheless, its mRNA (Figure 2a) and protein levels (Figure 2b, Figure S4a) were significantly reduced compared to control littermates (*Zfp36l2*^flox/flox^ C57/BL6). cfT4 and cfT3 were determined by ELISA at PND 30, 60, and 90. No major differences in cfT4 were detected in males compared to control littermates at any sampling time (data not shown). The t-*Zfp36l2*^-/-^ females manifested a trend towards decreased cfT4 levels at PND 30 and PND 60 (Figure S2), and the levels of cfT4 and cfT3 (Figure 1b,c, *n* = 6) were significantly reduced at PND 90. Concordantly with the hormone levels, the t-*Zfp36l2*^-/-^ females had reduced body weights compared to the controls (Figure 1d). Considering these data and the fact that females are more susceptible to hypothyroidism [37], only this sex was investigated further. H&E staining of thyroid sections did not demonstrate any major defects, although slightly smaller follicles were detected in the t-*Zfp36l2*^-/-^ females vs. controls (Figure S3).

**Figure 3:**
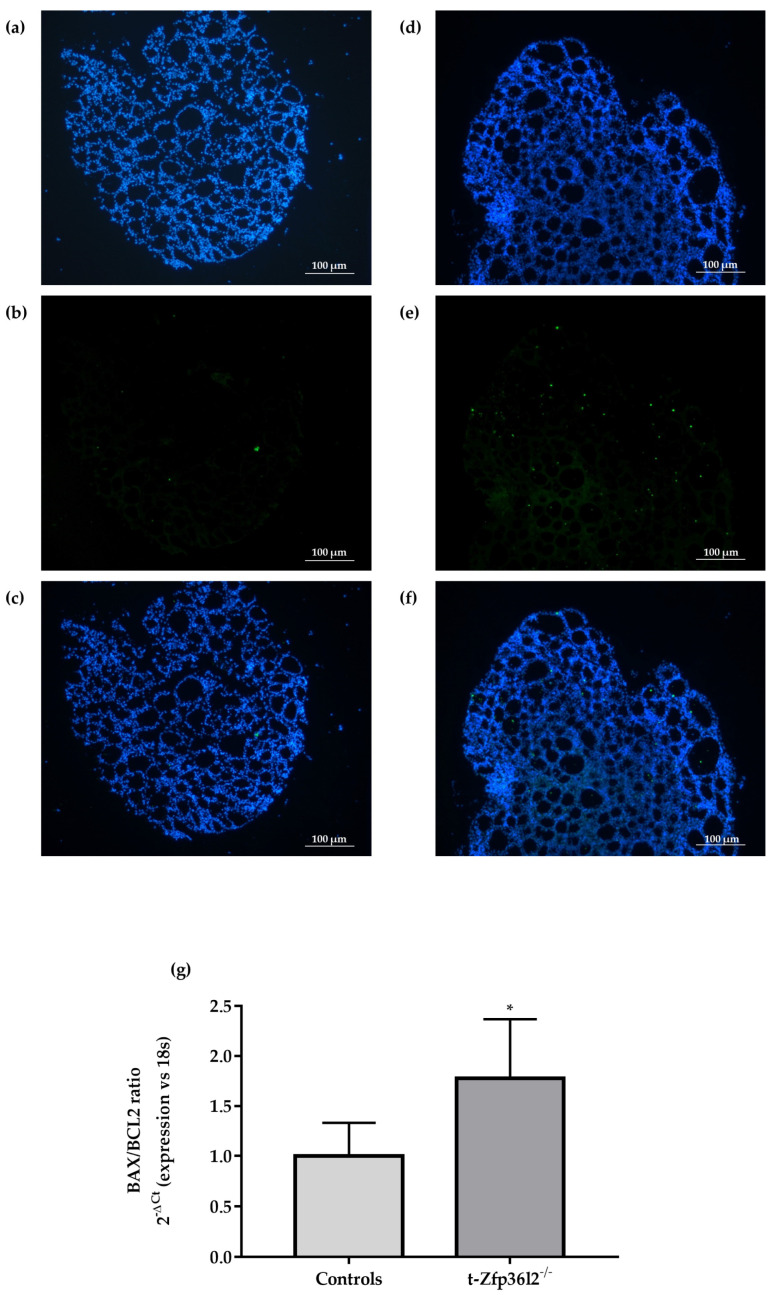
Analysis of apoptosis in control (*n* = 3) and t−*Zfp36l2^-/-^* mice (*n* = 3). 10X magnification images of DAPI staining of thyroid samples from controls (**a**) and t−*Zfp36l2^-/-^* mice (**d**); nuclei positive Tunel staining in controls (**b**) and t−*Zfp36l2^-/-^* mice (**e**). The merge of the previous images is shown in (**c**) for controls and (**f**) for t−*Zfp36l2^-/-^* mice. RTqPCR in extracts from thyroid samples was conducted to determine the levels of *Bax* and *Bcl2* mRNAs. The *Bax*/*Bcl2* transcript ratios in control (*n* = 3) and t−*Zfp36l2^-/-^* mice (*n* = 3) (**g**). RTqPCR *Bax*/*Bcl2* ratio between mean and standard deviation of *Bax* and *Bcl2* ∆Ct values (FC values of each gene vs. FC of 18SrRNA), in KO (*n* = 3) and control mice (*n* = 3); * *p* < 0.05.

To more completely characterize the thyroid phenotype, thyrocyte proliferation and apoptosis were investigated. No major differences in proliferation levels (Ki67 staining) were observed between the t-*Zfp36l2*^-/-^ and control females (data not shown). Furthermore, in order to detect the possible presence of apoptosis, we performed Tunel assay staining. In this way, we detected a slight increase of apoptotic nuclei (Figure 3a–f) in thyroids from t-*Zfp36l2*^-/-^ vs. control females. These data were associated with an increase in the ratio of *Bax* over *Bcl2* mRNA (Figure 3g).

**Figure 4:**
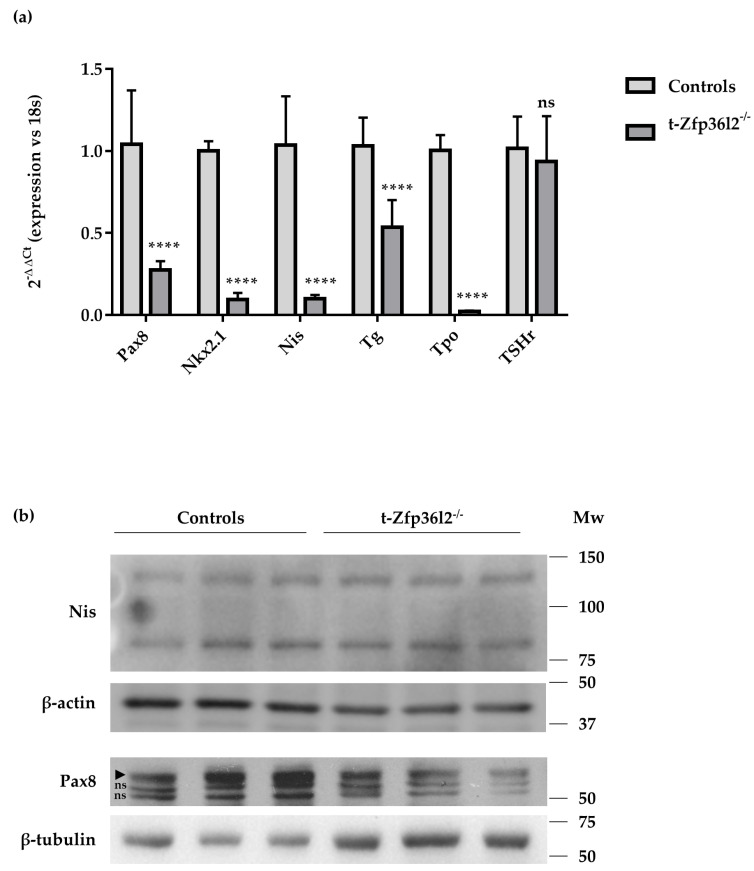
Analysis of thyroid-specific gene expression and protein levels in control and t-*Zfp36l2^-/-^* mice. (**a**) RTqPCR analysis of the expression of the thyroid-specific/enriched transcripts. (**b**) Western blotting analysis of NIS and PAX8 in extracts from thyroid samples of control (*n* = 3) and t−*Zfp36l2^-/-^* (*n* = 3) mice. In the Pax8 panel, arrowheads point to the specific band of Pax8; ns indicates non-specific bands. RTqPCR data are reported as mean ± standard deviation of ∆∆Ct values (FC values of gene normalized vs. FC values of 18SrRNA to obtain ∆Ct values of samples; ∆Ct values of KO samples are then normalized vs. ∆Ct of control samples to obtain ∆∆Ct values) in KO (*n* = 3) and control mice (*n* = 3); **** *p* < 0.0001.

We then investigated thyroid functionality by determining the expression levels of thyroid-specific transcripts (*Nis*, *Tg,* and *Tpo*) and transcripts for thyroid-enriched transcriptional factors (*Pax8*, *Nkx2.1*) by RTqPCR. These data demonstrated reductions of *Pax8*, *Nkx2.1*, *Nis, Tg,* and *Tpo* transcripts in t-*Zfp36l2*^-/-^ vs. controls (Figure 4a). The decreases in PAX8 and NIS levels were also demonstrated by Western blotting (Figure 4b, Figure S4b).

Collectively, the data show evidence of hypothyroidism in the t-*Zfp36l2*^-/-^ females and that the inactivation of *Zfp36l2* in the thyroid affects the function of the gland in more ways than its development and morphology, except for the slight increase in apoptosis detected in the t-*Zfp36l2*^-/-^ females.

### 2.2. Zfp36l2 Gene Inactivation Affects Expression of Key Thyroid Genes in Immortalized Thyrocytes

**Figure 5:**
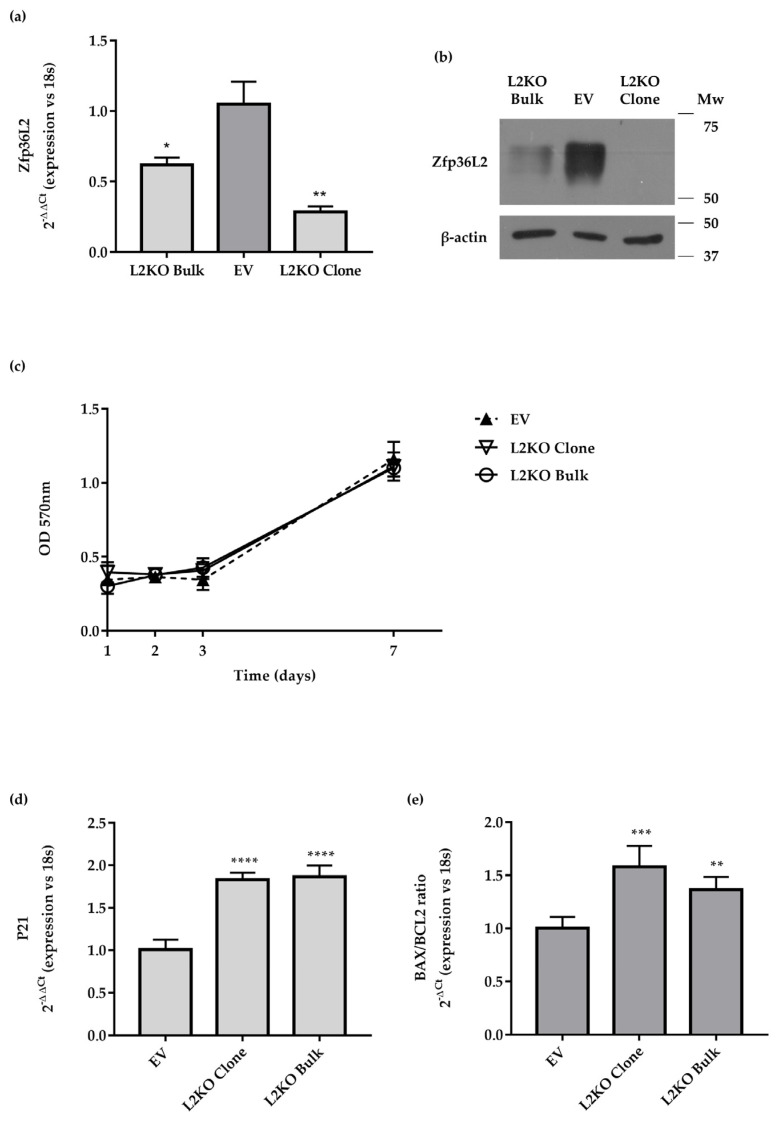
Analysis of the recombinant cell line FRTL5 *Zfp36l2* KO. Cells were generated as detailed in M&M by transducing them with lenticrispV2 empty (EV) or carrying a specific gRNA sequence targeting the *Zfp36l2* rat gene (L2KO). RTqPCR analysis (**a**) and Western blot (**b**) were conducted to control the knock-out efficiency both in the bulk culture and in one of the selected clones. (**c**) Cell growth of the L2KO cells, bulk culture and clone, and EV were monitored by MTT assay during 7 days of culture in normal medium. RTqPCR analysis of *P21* mRNA (**d**), *Bax*/*Bcl2* ratio of the L2KO cells, bulk culture, and clone, vs. EV (**e**). RTqPCR data are reported as mean ± standard deviation of ∆∆Ct values (FC values of gene normalized vs. FC values of 18SrRNA to obtain ∆Ct values of samples; ∆Ct values of KO samples are then normalized vs. ∆Ct of control samples to obtain ∆∆Ct values) in KO (*n* = 3) and controls (*n* = 3). RTqPCR *Bax*/*Bcl2* ratio between mean and standard deviation of *Bax* and *Bcl2* ∆Ct values (FC values of each gene *vs* FC of 18SrRNA), in KO (*n* = 3) and controls (*n* = 3). * *p* < 0.05; ** *p* < 0.005; *** *p* < 0.001; **** *p* < 0.0001.

To investigate the molecular mechanisms underlying the effects of *Zfp36l2* inactivation in thyrocytes, we generated a cellular model by knocking out *Zfp36l2* in immortalized rat thyroid cells (FRTL5). FRTL5 is a well-characterized in vitro model widely used to study thyroid function since it retains the ability to concentrate iodine, produce thyroglobulin, and respond to TSH stimulation [38]. The *Zfp36l2* knock-out FRTL5 cells (L2KO, from now on) were obtained by lentiviral delivery of the Crispr/Cas9 components, as detailed in M&M, whereas control cells were infected with an empty vector (EV, from now on). We determined the levels of the *Zfp36l2* mRNA and protein both in the bulk culture and in selected clones. A representative result is reported in Figure 5a, showing that *Zfp36l2* mRNA was strongly reduced and the protein was not detectable in the clone; whereas, they were both significantly reduced in the bulk culture (Figure 5a,b). We chose to work on the bulk culture as being more representative of the heterogeneity found in the in vivo model.

L2KO cells did not show any major changes in cellular morphology vs. EV cells. Since transcripts playing a role in the regulation of the cell cycle and apoptosis are targeted by ZFP36L2 in other cell types [39], we tested both cellular activities. MTT assays did not show any major differences in the proliferation of exponentially growing EV and L2KO cells (Figure 5c). Despite that, we found an increased expression of *P21/Waf1* (Figure 5d), suggesting an impairment of the cell cycle. Furthermore, the increased ratio of *Bax/Bcl2* transcripts pointed to a possible increase in apoptosis (Figure 5e).

**Figure 6:**
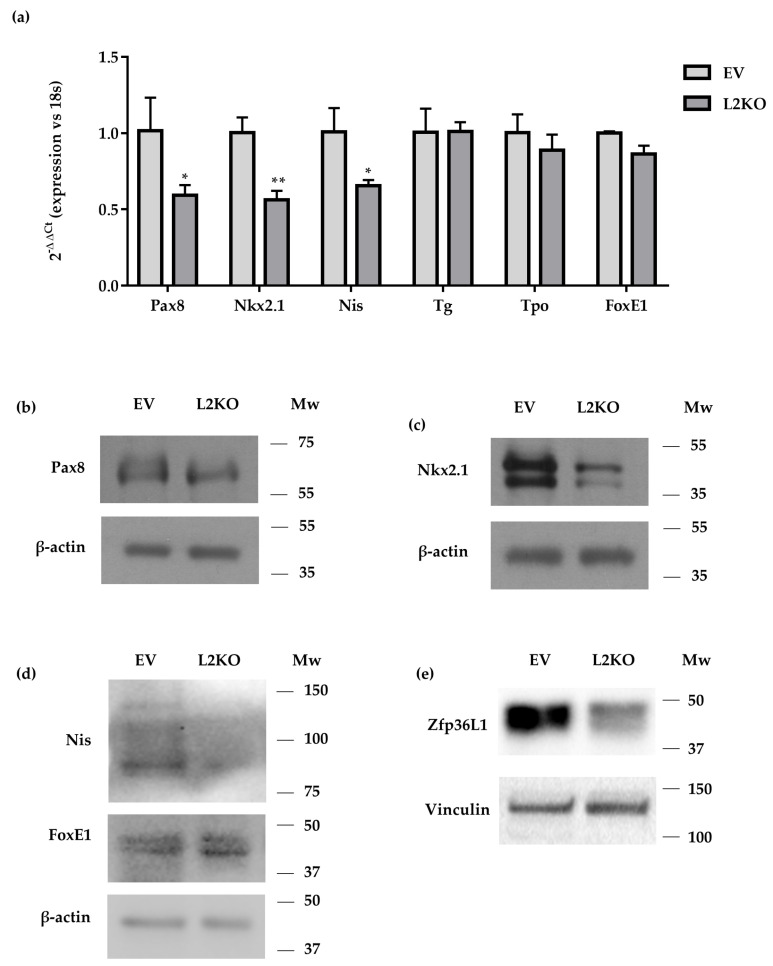
Analysis of thyroid-specific/enriched genes. Gene expression (**a**) or protein (**b**–**d**) in L2KO and bulk culture vs. EV cells. ZFP36L1 levels were also tested by Western blotting (**e**). RTqPCR data are reported as mean ± standard deviation of ∆∆Ct values (FC values of gene normalized vs. FC values of 18SrRNA to obtain ∆Ct values of samples; ∆Ct values of KO samples are then normalized vs. ∆Ct of control samples to obtain ∆∆Ct values) in KO (*n* = 3) and controls (*n* = 3). * *p* < 0.05; ** *p* < 0.005.

The molecular characterization of thyroid-specific or enriched transcripts and proteins conducted in exponentially growing EV and L2KO cells mainly agreed with what we found in mice. Indeed, *Pax8*, *Nkx2.1,* and *Nis* mRNAs were significantly reduced (Figure 6a), as were their encoded proteins (Figure 6b–d, Figure S5) in L2KO vs. EV. In contrast, no differences were observed for *Tg* and *Tpo* in the L2KO cells (Figure 6a) or for *Foxe1*/*TTF2* (Figure 6a,d, Figure S5). As members of the ZFP36 family might have redundant functions, we analyzed the cellular content of ZFP36L1 and ZFP36 in the L2KO cells. Levels of ZFP36L1 were reduced in L2KO vs. EV (Figure 6e, Figure S5), whereas *Zfp36* mRNA showed a slight trend towards a decrease (data not shown).

Overall, the data suggest a role for ZFP36L2 in thyrocyte functionality in rat immortalized thyrocytes.

### 2.3. Zfp36l2 Inactivation Impairs The FRTL5 Response to TSH 

**Figure 7:**
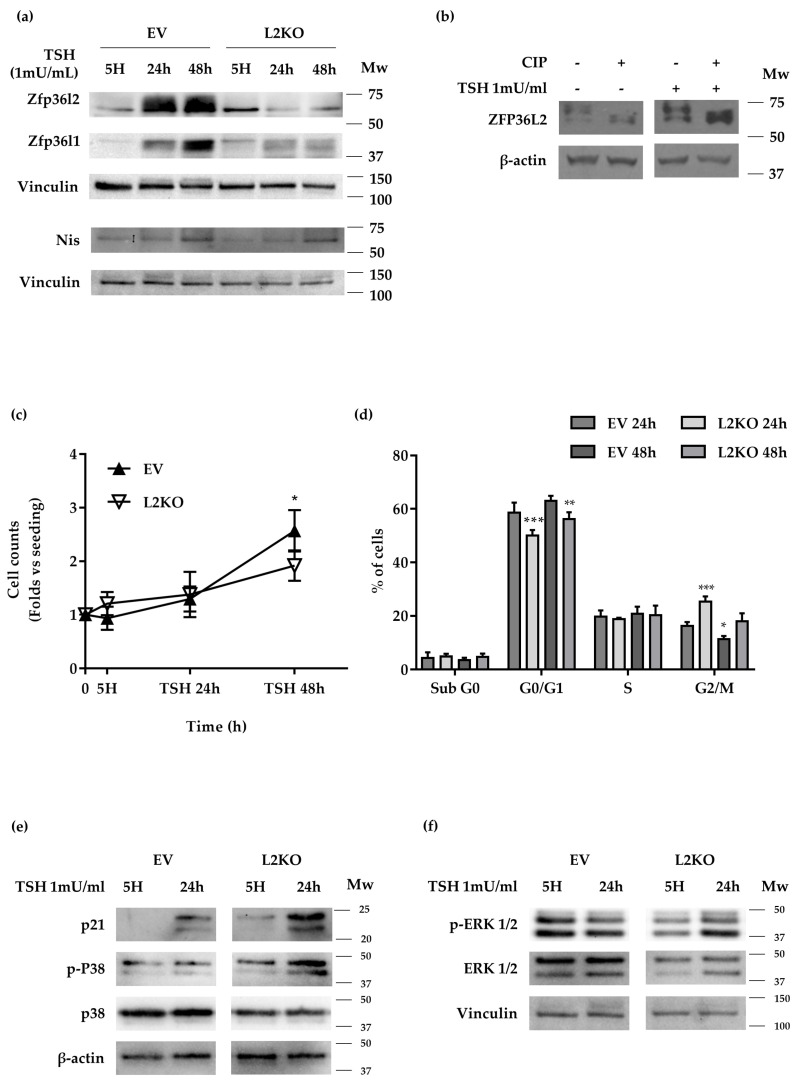
Characterization of the time-dependent response to TSH in L2KO and EV cells. Cells were TSH-starved and then stimulated for the reported time as detailed in M&M. (**a**) Western blotting analysis of ZFP36L2, ZFP36, L1, and NIS. (**b**) Analysis of the phosphorylation of ZFP36L2 after TSH induction and Calf Intestinal Phosphatase (CIP) treatment of the whole cell lysate. Absence of the ZFP36L2 signal after CIP treatment demonstrates that TSH induced protein phosphorylation. (**c**) Cell counting conducted on detached cells stained with trypan blue. The indicated time points on the - axis are, 0, number of cells at seeding time; 5H, number of cells after 3 days starvation; TSH 24 h, number of cells after 24 h of stimulation with TSH 1 mU/mL; TSH 48 h, number of cells after 48 h of stimulation with TSH 1 mU/mL. (**d**) Analysis of the cell cycle was conducted by FACS analysis using Propidium Iodide staining on cells stimulated with TSH for 24 h or 48 h. (**e**) Western blot analysis of P21, P38-MAPK, and (**f**) ERK 1/2-MAPK. The activation status of the last two is reported (p-P38 and p-ERK). Trypan blue exclusion cell counts and FACS data reported as mean ± standard deviation of 3 independent experiments. * *p* < 0.05; ** *p* < 0.01; *** *p* < 0.001.

Since TSH stimulates both cellular proliferation and the expression of thyroid-specific or enriched genes in FRTL5 cells [40], we investigated whether the loss of ZFP36L2 could affect these cells’ response to TSH. To test this, EV and L2KO cells were deprived of TSH by growing them in a medium without TSH (5H, from now) for three days. Such a procedure results in FRTL5 cell synchronization in the G0/G1 phase (Figure S6). After starvation, the cells were stimulated by adding a fresh complete growing medium (6H from now, containing 1 mU/mL TSH) for 24 and 48 h. We chose these time points since FRTL5 doubling time is reported to be 36 h [41]. First, we tested whether ZFP36L2 levels could be directly regulated by TSH. We found that ZFP36L2 protein levels were reduced after TSH starvation and increased after TSH stimulation (Figure 7a, Figure S7a). Interestingly, the levels of ZFP36L2 protein seemed to be increased in starved L2KO cells vs. EV (Figure 7, Figure S7). The same behavior was observed with ZFP36L1 (Figure 7a, Figure S7b). Furthermore, TSH appeared to promote ZFP36L2 protein phosphorylation since treatment of the extracts with calf intestinal phosphatase resulted in the appearance of a single band of immunoreactive protein (Figure 7b). To confirm TSH signaling, we analyzed NIS protein levels in L2KO and EV cells. As shown in Figure 7a, NIS was downregulated in starved L2KO cells compared to EV cells, the increase after TSH stimulation was lower, and it was delayed in L2KO cells (Figure S7c). Overall, these data suggested that the response to TSH was impaired in the absence of ZFP36L2.

Since TSH controls both proliferation and survival of thyrocytes, we investigated both processes in L2KO and EV cells treated as above. Cell counts showed a reduced number of L2KO vs. EV cells after 48 h of TSH stimulation (Figure 7c). FACS analysis of the cell cycle by PI staining showed an increased G2/M population in L2KO vs. EV cells at 24 h and 48 h (Figure 7d), suggesting a faster exit of the L2KO cells from the G0/G1 phase. The distribution in different phases of the cell cycle of L2KO and EV cells was similar at 24 h and 48 h, indicating a longer duration of the G2/M phase. Analysis of the sub G0/G1 population did not show any difference in apoptotic cells (Figure 7d). Consistently, we found an increase of P21/WAF1 content in L2KO vs. EV cells (Figure 7e, Figure S7d) after starvation and TSH stimulation. Increases in p-ERK1/ERK2 were detected in all conditions in L2KO vs. EV cells (Figure 7f, Figure S7f), despite the overall reduction of the ERK1/ERK2 proteins (Figure 7f, Figure S7f). Analysis of P38 MAPK activation showed an increase of p-P38 levels, already detectable in starved L2KO cells vs. EV (Figure 7f, Figure S7g), and even more evident after 24 h of TSH stimulation (Figure 7f, Figure S7g). Interestingly, the levels of total P38α protein showed a reduction in L2KO vs. EV (Figure 7f, Figure S7h). The increased p-P38 in L2KO cells vs. EV was already apparent after shorter periods of TSH stimulation (Figure S8).

### 2.4. ZFP36L2 Modulates Thyrocyte Function and Apoptosis via the Notch1 Pathway In Vitro and In Vivo

**Figure 8:**
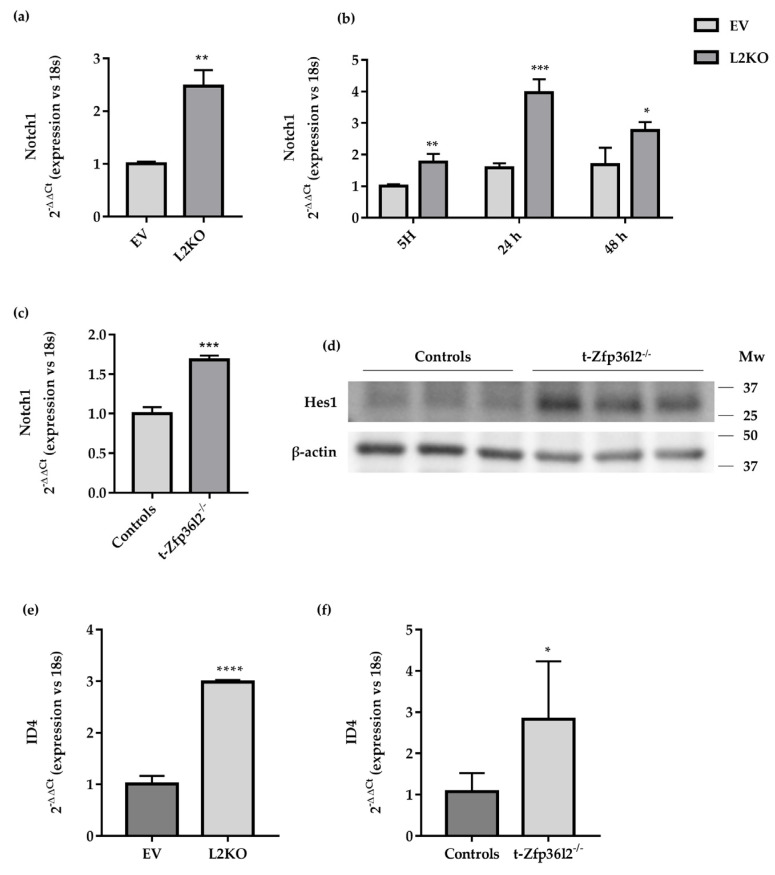
Modulation of *Notch1* pathways in t-*Zfp36l2^-/-^* mice and L2KO cells. (**a**) RTqPCR analysis of Notch1 expression in L2KO and EV cells and (**b**) RTqPCR analysis of Notch1 expression and its modulation by TSH signaling in L2KO and EV cells; 5H, cell culture medium deprived from TSH; 24 h and 48 h, time points of TSH signalling induction using complete cell culture medium. (**c**) RTqPCR analysis of Notch1 expression in thyroid extracts from t−*Zfp36l2^-/-^* (*n* = 3) and control (*n* = 3) mice. (**d**) Western blotting analysis of HES1 in extracts from thyroid samples of t−*Zfp36l2^-/-^* (*n* = 3) and control mice (*n* = 3). (**e**) RTqPCR analysis of *Id4* mRNA in L2KO and EV cells, (**f**) thyroid extracts from t−*Zfp36l2^-/-^* (*n* = 3) and control (*n* = 3) mice. RTqPCR data are reported as mean ± standard deviation of ∆∆Ct values (FC values of gene normalized vs. FC values of 18SrRNA to obtain ∆Ct values of samples; ∆Ct values of KO samples are normalized vs. ∆Ct of control samples to obtain ∆∆Ct values) in KO (*n* = 3) and controls (*n* = 3); *p < 0.05; ** *p* < 0.01; *** *p* < 0.001; **** *p* < 0.0001.

As described above, *Zfp36l2* gene inactivation reduces the levels of thyroid-specific transcripts as well as those of *Zfp36l1* both in vivo and in vitro. This observation suggests that thyroid-specific transcripts are not direct targets of ZFP36 protein activity. Another potential mechanism by which ZFP36L2 could modulate the levels of thyroid-specific transcripts was described in T-lymphoblastic leukemia, in which ZFP36L2 and L1 were able to modulate the cellular content of *Notch1* mRNA and protein [42], a signaling pathway involved in thyrocyte function [15,43,44]. Therefore, we assayed the levels of the *Notch1* gene in our experimental settings in vitro and in vivo. *Notch1* gene expression was increased in exponentially growing L2KO vs. EV cells (Figure 8a, protein is shown in Supplementary Figure S9a), as well as in TSH-starved cells (Figure 8b, protein is shown in Supplementary Figure S9b). Furthermore, TSH stimulation increased its levels in EV after 48 h, whereas it had little effect in L2KO cells (Figure 8b, protein is shown in Supplementary Figure S9b). This was also in agreement with a reduced ARE binding activity of ZFP36L2 when phosphorylated [45]. This result was confirmed in thyroids from t-*Zfp36l2*^-/-^ mice. Indeed, an increase of *Notch1* gene expression (Figure 8c, protein shown in Supplementary Figure S9c) was detected in t-*Zfp36l2*^-/-^ vs. controls and was further supported by the induction of HES1, a direct NOTCH1 target [46] (Figure 8d, Supplementary Figure S9c).

NOTCH1 and HES1 are positive regulators of the expression of thyroid-specific genes that decreased following *Zfp36l2* gene inactivation. We looked for other NOTCH1 targets that could act as transcriptional repressors. Since ID4, a transcriptional repressor, is a target of NOTCH1 [47] and has been reported as a negative regulator of PAX8 transcriptional activity [48], we attempted to verify its increase in our models. We found, by RTqPCR, that levels of the *Id4* transcript were increased in L2KO vs. EV cells (Figure 8e) as well in thyroids from t-*Zfp36l2*^-/-^ mice vs. controls (Figure 8f).

Collectively, those data suggest that ZFP36L2 might exert its effects by deregulating NOTCH1 signaling and promoting an increase of HES1 and *Id4* levels in thyrocytes. This imbalance might damage thyrocyte function and survival.

## 3. Discussion

Tissue-specific gene expression results from the interplay between transcriptional and post-transcriptional mechanisms. The former has been extensively investigated; the latter is almost unexplored in thyroid development and function. Studies of post-transcriptional regulation in the thyroid could improve our knowledge of the genes altered in CH, whose mechanisms of action are often unexplained. It is noteworthy that several recent papers have described the role of splicing modulation in thyroid development in vivo [49], suggesting this mode of post-transcriptional regulation as a mechanism involved in thyroid development, function, carcinogenesis, and thyroid hormone signaling [19]. Hence, the search for novel alleles of CH candidate genes continues since their interaction with already characterized genes involved in CH, and with environmental factors, could play a role in the pathogenesis of CH. 

In our previous work, we observed that levels of the mRNA of ZFP36L2, a protein that can promote RNA degradation [50], were increased during thyroid development [32] and reduced during hypothyroidism promoted by environmental stressors [34], as well as during thyroid carcinogenesis [33,41]. Its role in thyroid development and function has not yet been characterized.

Overall, the results reported here confirm the role of the ZFP36L2 protein in thyroid function. Indeed, we report that *Zfp36l2* gene inactivation promotes thyroid dyshormonogenesis, as suggested by reduced levels of cfT4 in the mouse model and normal thyroid development and morphology. We also report a slight impact on the survival of thyrocytes in vitro and in vivo. This effect appears to be mediated by the altered expression of thyroid-specific enzymes and their transcriptional regulators observed both in vivo and in vitro. Mice knocked-out for *Zfp36l2*^-/-^ generally die before weaning and exhibit a marked reduction of fetal liver hematopoietic progenitors [29]. In our previous work, we suggested that the ZFP36L2 reduction promoted by environmental factors might contribute to the observed pancytopenia by promoting hypothyroidism [34]. This suggestion is supported here by the reduction of cFT4 in the *Zfp36l2*^-/-^ mice. It is noteworthy that decreases in cfT4 and cFT3 levels have been considered determinants of the defect in erythropoiesis detected in TRα knockout mice at later life stages [51]. Furthermore, the detected hypothyroidism might also contribute to ZFP36L2′s influence on ovulation and oocyte maturation. Indeed, hypothyroidism might affect both processes [52]. Therefore, the hypothyroidism detected in the *Zfp36l2*^-/-^ females might be a causative factor in several of the phenotypes associated with ZFP36L2 activity loss.

In oocytes, it was shown that PKA inhibition significantly rescued the effects associated with *Zfp36l2* inactivation [53]. Indeed, PKA, as well as other signaling pathways, are activated by TSH in the thyroid [54]. In agreement with these previously shown data, we describe different responses to TSH in EV and L2KO cells. Indeed, ZFP36L2 is apparently phosphorylated after TSH stimulation, and its levels are increased. Along with the inhibition of its activity, we also report an increase in cleaved NOTCH1 protein, one of its targets in other tissues [45]. We focused our attention on the *Notch1* pathway since its role in thyroid development and function has been reported previously. For example, it has been shown that *Notch1* pathway activation strongly influences thyroid development if induced before thyroid cell specification in zebrafish [15]. In our in vivo models, such an effect was only partially confirmed, given that no defects were detected in gland development [15]. We suggest that this might depend on the later expression of *Pax8-Cre,* which could drive *Zfp36l2* gene inactivation at later stages of thyroid development.

The NOTCH1 downstream target Hes1 has also been implicated in thyroid development and function [43] since its genetic inactivation resulted in the reduction of NKX2.1 positive cells during thyroid development and a reduced expression of thyroid-specific genes [44]. We demonstrated increases in cleaved-NOTCH1 in the t-L2KO thyroids, but also an impairment of thyroid function like that described in *Hes1*^-/-^ mice. This discrepancy might be due to the window of analysis since *Hes1*^-/-^ mice died at embryonic stage E. 16.5; thus, the effects at later life stages might be different. Furthermore, NOTCH1 targets several genes that might play other roles in regulating gene expression in the thyroid, meaning that some of them could affect the expression of thyroid-specific enzymes, while others could influence the expression of proteins able to modulate proliferation and survival of thyrocytes [44]. Indeed, activated *Notch1* pathways have been implicated in the delayed cell cycle progression and apoptosis seen in FRTL5 [42]. Thus, its activation could be involved in the reduction of cell proliferation seen in L2KO with the increase in apoptosis seen in thyroids from *Zfp36l2*^-/-^ female mice. Furthermore, NOTCH1 pathway activation has been associated with the increase of P21 in thyroid cancer models [55]. The expression profile of thyroid-specific transcripts is in contrast with previously published data demonstrating that NOTCH1 was their positive transcriptional regulator. This implies that NOTCH1 activation along the ZFP36L2-NOTCH1 axis in the thyroid might involve other players. We propose that one of them could be the ID4 protein, a transcriptional repressor since it is induced by NOTCH1 and could inhibit PAX8-regulated gene expression by interacting with PAX8 protein [48]. Consistently, we detected *Id4* mRNA increases in thyrocytes carrying inactivated *Zfp36l2* and decreases in *Pax8* transcript levels since PAX8 can also regulate its own promoter [48]. Overall, the data suggest that different NOTCH1 targets are involved in the control of cell proliferation (HES1) and thyroid-specific gene expression (ID4). Furthermore, considering the modulation of epigenetic mechanisms by ZFP36L2 [35], we cannot rule out their involvement. Indeed, a Notch Repressive Complex (NRC) containing NuRD and PRC1 has been described. PRC1 is a target of ZFP36 [56,57] and could increase following the deletion of *Zfp36l2* and inhibit the expression of *Pax8* and *Nkx2.1*, as reported in other systems [58].

Interestingly, we also found evidence for the hyper-activation of the P38-MAPK pathway after TSH stimulation in L2KO vs. EV cells. This might depend on the accumulation of MKK6 protein (its upstream activator), whose transcript levels are modulated by mRNA decay mechanisms [59]. This suggestion would agree with the increase of *Nis* mRNA in L2KO, already described as dependent on CHOP activation in thyroid cells [60]. The increased activation of the P38-MAPK pathway might contribute to slowing down the G2/M transition in L2KO and to the increased apoptosis in vivo since it is inhibited in thyroid hyperplastic and neoplastic tissues [61]. In our models, this event is driven by ZFP36L2 inactivation in association with the reduced expression of thyroid-specific transcripts. This last is a hallmark of the transformation of thyrocytes [33]. Finally, apoptosis is an early and pivotal step in thyroid carcinogenesis, and ZFP36L2 inactivation results in its increase. Hence, ZFP36L2 might participate in the generation of surviving thyrocytes that are more prone to transformation [24,62]. 

Overall, the data suggest that mRNA decay is a mechanism for regulating thyroid function by modulating thyroid-specific gene expression. The silencing of ZFP36L2 by gene inactivation indirectly modulates the expression of thyroid-specific enzymes and their transcriptional regulators. These events promote hypothyroidism in vivo, beginning at the early phases of post-natal life. The data support the hypothesis that ZFP36L2 exerts this activity by regulating the NOTCH1 pathway, including its targets HES1, active on modulation of proliferation and apoptosis, and ID4, modulating thyroid-specific gene expression. Further studies are needed to characterize both mechanisms. Finally, the similarity of the in vivo and in vitro results confirm the suitability of the FRTL5 cells as an appropriate in vitro model to investigate the mechanisms regulating thyrocyte function by in vitro gene-targeting approaches.

## 4. Materials and Methods

### 4.1. Plasmids and Antibodies

lentiCRISPR v2 was a gift from Feng Zhang (Addgene plasmid #52961; http://n2t.net/addgene:52961; RRID: Addgene_52961); psPAX2 was a gift from Didier Trono (Addgene plasmid #12260; http://n2t.net/addgene:12260; RRID: Addgene_12260); pMD2.G was a gift from Didier Trono (Addgene plasmid #12259; http://n2t.net/addgene:12259; RRID: Addgene_12259).

The antibodies used are listed below:

- Anti ZFP36L2 (*PAB17366*) from Abnova
- Anti ZFP36L1/L2 (*2119*), anti P21 (*2947*), anti ERK 1/2 (*9102*), anti Cleaved-NOTCH1 (*4147*), and anti β-ACTIN (*3700*) from Cell signalling
- Anti VINCULIN (*V4139*) from Merk
- Anti β-Tubulin (*sc-5274*), Anti HES1 (*sc-166410*), anti p-P38 (*sc-7973*), anti P38α/β (*sc-7972*), anti p-ERK 1/2 (*sc-7383*), anti Rabbit (*sc-2357*) from Santa Cruz
- Anti Mouse (*G21040*) from Thermo Fisher
- Anti NIS [63], PAX8 [64], NKX2.1 [64], FOXE1 [65]-homemade

### 4.2. Animals

Mice were housed in the Biogem Animal mice facility (AMF) at 22 ± 2 °C under a 12 h dark/light cycle. Mice received standard rodent pelleted chow 4RF21 (Mucedola, Settimo Milanese, Italy) and water ad libitum. All animal experiments were performed in accordance with guidelines approved by the Italian Ministry of Health (889/2017-PR; 20 November 2017). The number of mice enrolled in the study was established through a G-Power analysis, required in preparing the documents to obtain the authorization from the Italian Ministry of Health, to work with animal models (α = 0.01; 1-β = 0.85; δ = 4.12). To obtain *Pax8-Cre-Zfp36l2*^-/-^ mice, we crossed heterozygous C57/BL6 mice carrying *Cre* recombinase cDNA in the *Pax8* locus [36] with C57/BL6 mice carrying the *Zfp36l2* locus with floxed exon 2 in order to obtain *Pax8*-*Cre*-*Zfp36l2^+^*^/-^. Heterozygous mice *Pax8*-*Cre*-*Zfp36l2*^+/-^ were further mated to generate *Pax8-Cre-Zfp36l2*^-/-^ mice. Genotypes of the recombinant mice were assessed by PCR analysis. Genomic DNA was extracted as previously described [66] by ear clip biopsies, and genotypes were determined by PCR:

*Zfp36l2*-Forward primer: AGA TAC CAA ACA CTT AGG TCT CAG ATG AG; Reverse primer: ACC ACC ACA AAG GAG GCT GAG A. WT allele 276 bp; Floxed allele 418 bp;
*Pax8*-*Cre* PCR was performed as previously indicated [36].

### 4.3. Cells

Rat immortalized thyrocytes were maintained in Coon’s modified F12 medium (Merk, Darmstadt, Germany) supplemented with 5% Newborn Bovine Serum, NCS (HyClone Laboratories, Logan, UT, USA), 1% penicillin/streptomycin (Thermo Fisher Scientific, Carlsbad, CA, USA), and six hormones, including 1 mU/mL TSH (Merck). L2KO and EV cells were generated by lentiviral infection, as previously described [67], using CRISPR/Cas9 technology, based on a construct already available in the laboratory. Briefly, Human Embryonic Kidney 293T cells, HEK293T, were maintained in DMEM Complete medium (EuroClone, Pero (MI), Italy) supplemented with 10% Fetal Bovine Serum, FBS (Merk), 1% Penicillin/Streptomycin (Gibco), and 1% l-glutamine (Gibco), and used as packaging cells for lentiviral production. HEK293T cells were transfected by calcium-phosphate, using a DNA mix containing lentiCRISPRv2 (10 µg) harboring or not anti-rat-Zfp36L2 specific gRNA and psPAX2 (10 µg) and pMD2.G (1 µg) as packaging vectors. Forty-eight hours post-transfection, cell supernatants were collected, filtered through a 0.45 μM filter, and 20 μg/mL of polybrene (Merk) was added. FRTL5 cell media was then replaced with infective media, and cells were incubated for 6 h. Later, the infective cell medium was replaced with normal Coon’s F12 medium and, after an additional 24 h, 1 μg/mL of puromycin was added to start selection.

### 4.4. MTT Assay and Cell Counts

MTT assays were performed as previously described [67]. Briefly, 5 × 10^3^ cells were plated in 96 multi-well plates. The cells were allowed to grow for up to 7 days. At the indicated time points, 500 µg/mL MTT was added to each well for 1 h. After incubation, samples were resuspended in 100% DMSO, and absorbance was read at 570 nm using an Envision 2103 Multilabel Reader (Perkin Elmer, Waltham, MA, USA).

For cell counts, 1 × 10^5^ cells were plated in 12 multi-well plates. One day later, cells were starved using a medium lacking TSH (5 hormones media, 5H) for 3 days. Next, the 6H medium was added. Cells were harvested at the indicated time points and counted using a TC20 cell counter (Biorad, Hercules, CA, USA) and trypan blue exclusion (seeding was considered as Time 0).

### 4.5. Cell Cycle Analysis, Real-Time PCR, Western Blotting, and CIP Treatment

Cells were plated in plates (10 cm) at 3.5 × 10^4^ cells/cm^2^ the day before the experiments. The next day, 5H medium was added to the cell for TSH deprivation. TSH stimulation was achieved by adding cell culture medium containing 1 mU/mL TSH (6 hormones media, 6 h). Cells were harvested before replacement of the medium (time 0) and after 24 and 48 h. The collected cells were in part fixed in 70% ethanol for PI staining and FACS analysis [53], in part lysed in TRIZOL reagent (Thermo Fisher Scientific) for analysis of mRNA levels, and, finally, in part lysed in RIPA buffer (20 mM Tris-HCl pH 7.5, 150 mM NaCl, 1 mM Na2EDTA, 1 mM EGTA, 2% NP-40, 1% sodium deoxycholate, complete mini EDTA-free protease inhibitor cocktail-Roche-and PhosSTOP phosphatase inhibitor cocktail-Roche) for Western blotting analysis.

Reverse transcription-quantitative PCR (RTqPCR) experiments were conducted as previously reported [68]. First, RNA was converted to cDNA using Quantitect Reverse Transcription Kit (Qiagen, Hilden, Germany) [68]. Subsequently, mRNA levels were evaluated as previously described [69], using the PowerUP SYBR green Master Mix (Thermo Fisher) and primers listed in table S1.

Whole-cell lysates for Western blotting analysis were prepared and quantified using Bradford reagent (Biorad). After BIS-TRIS SDS-PAGE, proteins were transferred to 0.2 µm PVDF membranes (Thermo Fisher Scientific). Membranes were blocked with 5% non-fat dry milk (Biorad) and incubated overnight at 4 °C with the indicated antibodies. Signals were revealed using ImmunoCruz Western blotting luminol reagent (Santa Cruz Biotechnology, Dallas, TX, USA) or Immobilon ECL Ultra (Merk) and acquired using a Molecular Imager Chemidoc XRS+ and Image Lab software (Biorad), or Kodak Medical X-ray Processor 2000 and Fujifilm Super RX-N films. Protein signal quantification was performed using Fiji NIH Image J software. For the analysis of ZFP36L2 phosphorylation, whole-cell lysates (30 µg) were treated with 30 units of Calf intestinal phosphatase (CIP, New England Biolabs, Ipswich, MA, USA) for 30 min at 37 °C before being loaded on gels for SDS-PAGE and Western blotting.

### 4.6. Thyroid Tissue Preparation

Thyroids from 6 female mice, aged 3 months, of each genotype (*Zfp36l2^flox^*^/flox^ and *Pax8cre-Zfp36l2* ^flox/flox^) were sampled by dividing the right and left lobes. Next, samples were divided as follows: 6 lobes were formalin-fixed and paraffin-embedded; 3 lobes were lysed in TRIZOL reagent for gene expression analysis, as described before; and 3 lobes were lysed in RIPA buffer for Western blotting analysis, as described before. Lysis of the tissue samples was performed using a Misonix XL2020 sonicator, at the power setting 4, for 15 s, on ice.

### 4.7. Measurement of fT3 and fT4 Hormones

FT4 and fT3 levels were measured in 6 serum samples from 3-month-old female animals of each genotype by using an ELISA kit (Diametra, Roma, Italy) following the manufacturer’s instructions, as previously described [68], and reading at the Biotek ELX800 multi-plate reader.

### 4.8. Histology and Tunel Assay

Hematoxylin and Eosin staining were performed on 5 µm tissue slices prepared from 6 samples of Formalin-fixed and Paraffin-embedded thyroids from 3-month-old female animals of each genotype, as previously described [33].

Tunel assays were performed on 5 µm tissue slices prepared from 3 samples of Formalin-fixed and Paraffin-embedded thyroids from 3-month-old female animals of each genotype using In Situ Cell Death Detection Kit, Fluorescein (Roche, Basel, Switzerland), following the manufacturer’s instructions. Images were acquired using a Zeiss Axioplan 2 microscope at 10X magnification and ZEN 3.1 software.

### 4.9. Statistical Analysis

Graphs and statistical analysis were performed using GraphPad 7 (Prism). Unpaired Student’s *t*-test (for direct comparisons) or Two-way ANOVA (for multiple comparisons) were performed to evaluate the significance of differences.

## Supplementary Materials

The following are available online at https://www.mdpi.com/article/10.3390/ijms22179379/s1, Table S1. List of primers used for RTqPCR. Figure S1. Hematoxylin and Eosin staining of the thyroid of *Zfp36l2^-^*^/-^ mice (*N*= 4, a,a^1^) and controls (*N* = 5, b,b^1^) at PND21. a,b images are 10× magnification, a^1^,b^1^ images are 40X magnification. Figure S2. ELISA quantification of cfT3 and cfT4 in 1-month- and 2-month-old mice. Controls (*N* = 6) and t-*Zfp36l2^-^*^/-^ (*N* = 6) mice were used. Figure S3. Haematoxylin and eosin staining of the controls (*N* = 3) and t-*Zfp36l2^-^*^/-^ (*N* = 3) thyroids. No appreciable morphological changes are evident (10× magnification). Figure S4. Densitometric analysis of Western blotting experiments shown in Figure 3. Figure S5. Densitometric analysis of Western blotting experiments shown in Figure 5. Figure S6. Cell cycle analysis of L2KO vs. EV cells to verify the efficiency of 3 days of TSH deprivation. Cells were starved as detailed in M&M. The data show that about 90% of L2KO and EV cells were in the G0/G1 phase, as expected. Figure S7. Densitometric analysis of Western blotting experiments shown in Figure 6. Figure S8. Analysis by Western blotting of the activation of P38-MAPK pathways at early times of TSH stimulation. EV and L2KO were starved and stimulated with TSH as described in M&M. At the reported time points; the cells were collected, lysed in RIPA buffer, and analyzed by Western blotting (a). Densitometric analysis (b). Figure S9. Western blot analysis of cleaved-Notch1 in experimental points showed in Figure 8. Figure S10. Densitometric analysis of western blotting experiments showed in Figure S9.
