# Supplementary material for: ZFP36L2 Role in Thyroid Functionality
Source: Int J Mol Sci. 2021 Aug 29;22(17):9379. doi: 10.3390/ijms22179379 (PMC8431063; doi:10.3390/ijms22179379)
Supplement: Supplementary file 1 [file ijms-22-09379-s001.zip › ijms-1218201-supplementarfy.pdf]

International Journal of Molecular Sciences

Supplementary File

Table S1. List of primers used for Real Time PCR experiments.

Gene	Forward primer (5′-3′)	Reverse primer (5′-3′)
Rat *Zfp36l2*	TGATGTGAAGAGGCCAGGTC	CAAAGCCGGGAGACTTTCCT
Rat *P21*	CTGACGCCCTTCACCGCGAG	GCGAAGTCAAAGTTCCACCG
Rat *Bax*	TGCTAGCAAACTGGTGCTCA	TAGGAAAGGAGGCCATCCCAG
Rat *Bcl2*	CTGACGCCCTTCACCGCGAG	CAAAGGCATCCCAGCCTCCGT
Rat *Pax8*	GGCCACCAAATCTCTGAGCC	TGGGAATCGATGCTCAGTCG
Rat *Nkx2.1*	TGCTTTATGGTCGGACCTGG	GTCTTTGCGGAGGGTAGAGG
Rat *Nis*	TTGTGGTAATGCTCGTTGGC	TCACACCGTACATGGAGAGCC
Rat *Tg*	TGGTACCAAAAGCCTGCTGTG	GAGTCTGCCAGGAGTCCAAA
Rat *Tpo*	CCCTTTCCTCCTGAGAAGCAG	TCCCCTTTTCTTGAGGTTTCTTTTC
Rat-Mouse *Id4*	GAGCATTGGCGACGTTGTTT	GAGAAAAAGTTCCCCGCCCT
Rat *Notch1*	TGCTCTGCCTAACGCTGC	CTCCGCTGCAGACACAGG
Rat-Mouse *18SrRNA*	GGGCCTCGAAAGAGTCCTGT	CGGCTACCACATCCAAGGAA
Mouse *Zfp36l2*	CCCTCGCCCGTTATTCATCT	CCAGGGATTTCTCCGTCTTG
Mouse *Zfp36l1*	TGTCCGCCACCATTTTTCAC	GCTGGGAGTGCTGTACTTGA
Mouse *Pax8*	GCCATGGCTGTGTAAGCAAGA	GCTTGGAGCCCCCTATCACT
Mouse *Nkx2.1*	CGCCTTACCAGGACACCAT	GCTCGAGCTCGTACACCTG
Mouse *Nis*	TCCACAGGAATCATCTGCACC	CCACGGCCTTCATACCACC
Mouse *Tg*	CATGGAATCTAATGCCAAGAACTG	TCCCTGTGAGCTTTTGGAATG
Mouse *Tpo*	CAAAGGCTGGAACCCTAATTTCT	AACTTGAATGAGGTGCCTTGTCA
Mouse *Tshr*	TCCCTGAAAACGCATTCCA	GCATCCAGCTTTGTTCCATTG
Mouse *Notch1*	GCCGCAAGAGGCTTGAGA	GATTGGAGTCCTGGCATCGT

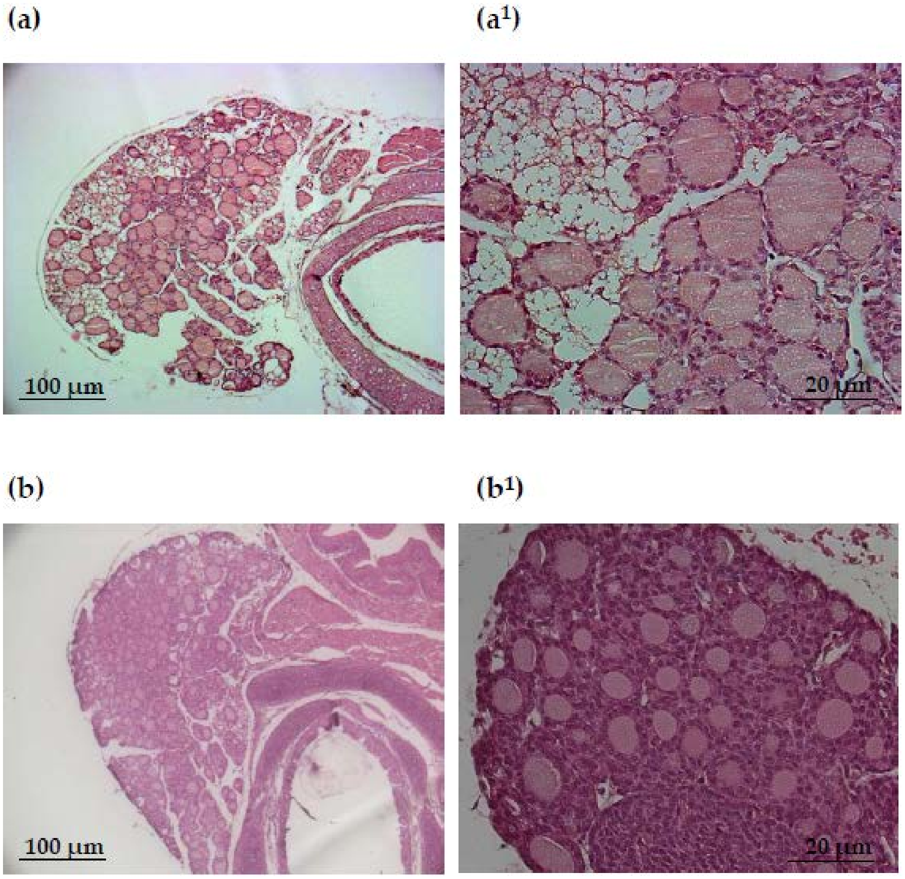

Figure S1. Hematoxylin and Eosin staining of the thyroid of *Zfp36l2-/-* mice (*N*= 4, **a**,**a**^{1}) and controls (*N* = 5, **b**,**b**^{1}) at PND21. **a**,**b** images are 10× magnification, **a**^{1},**b**^{1} images are 40× magnification.

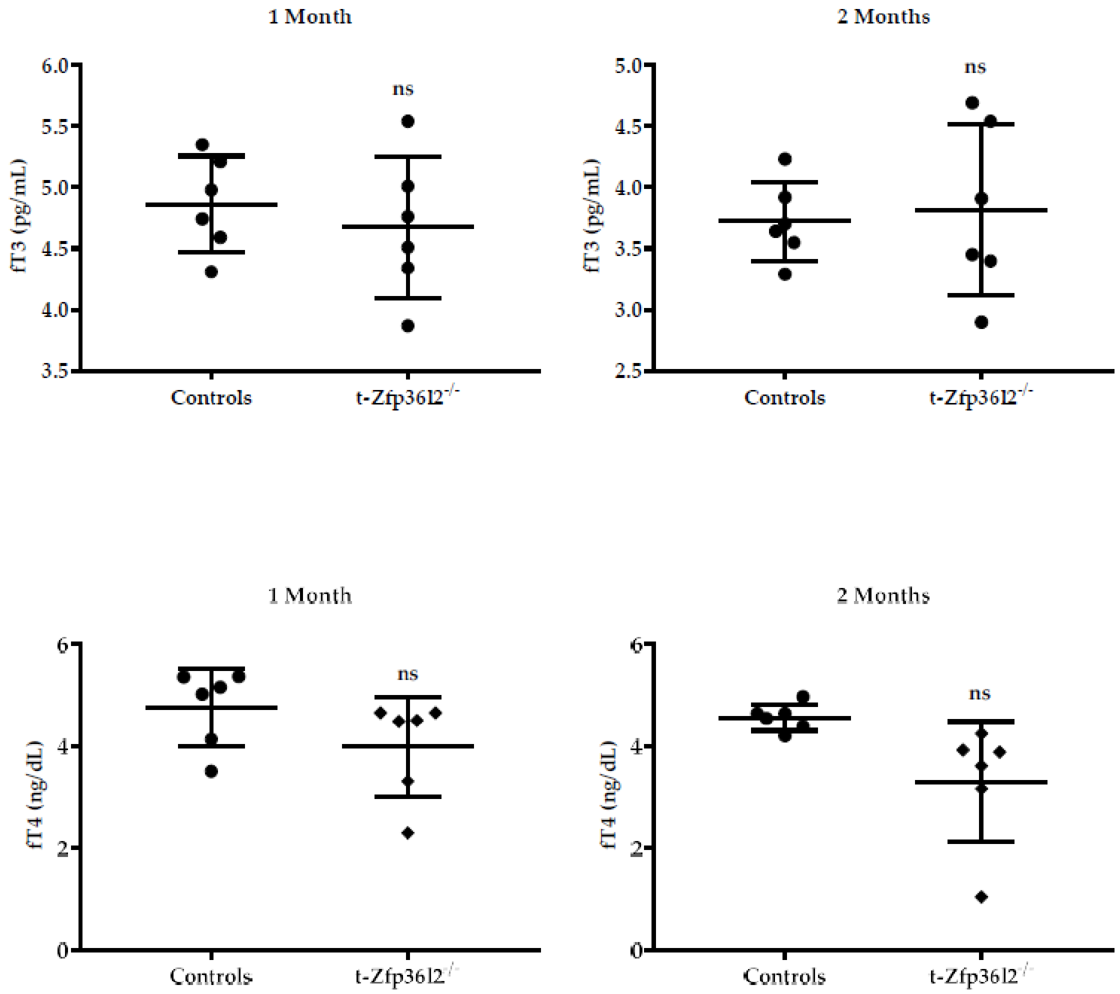

Figure S2. ELISA quantification of cfT3 and cfT4 in 1-month- and 2-month-old mice. Controls ($N = 6$) and t-*Zfp36l2*$^{-/-}$ ($N = 6$) mice were used. Data are reported as mean ± standard deviation. ns is for not significant.

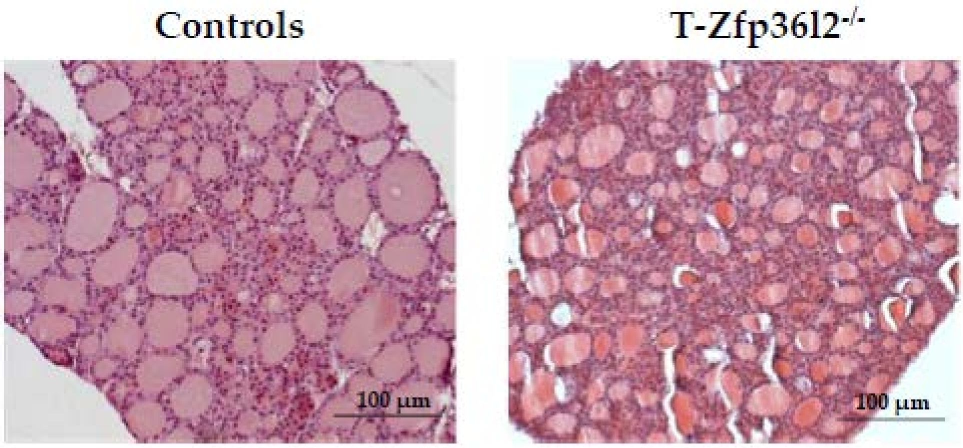

Figure S3. Haematoxylin and eosin staining of the controls ($N = 3$) and t-*Zfp36l2*$^{-/-}$ ($N = 3$) thyroids. No appreciable morphological changes are evident (10× magnification).

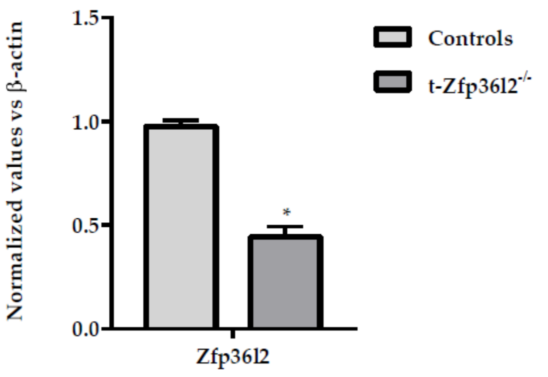

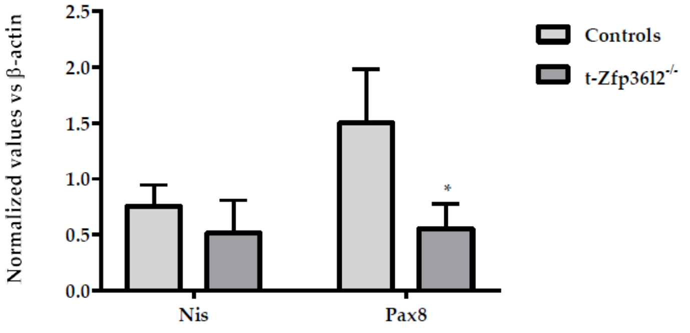

(**a**) (**b**)

Figure S4. (**a**) Densitometric analysis of Western blotting experiments shown in Figure 2b. *$p < 0.05$ (**b**) Densitometric analysis of Western blotting experiments shown in Figure 4b. * $p < 0.05$.

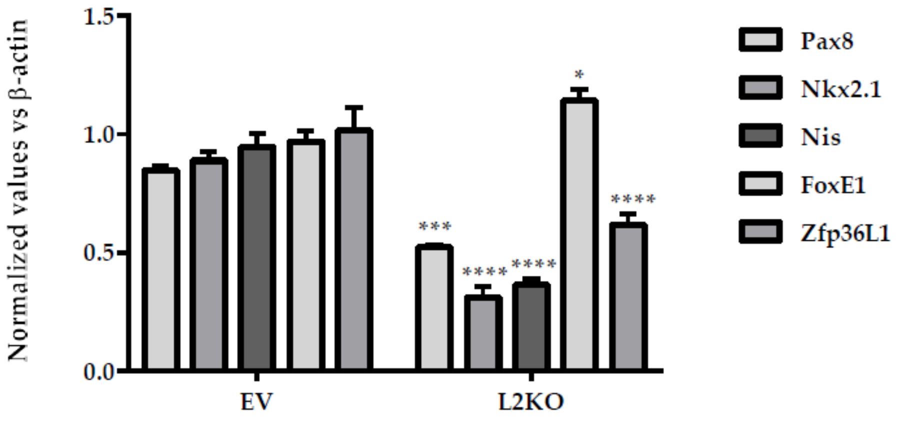

Figure S5. Densitometric analysis of Western blotting experiments shown in Figure 6b-e. *$p < 0.05$, ***$p < 0.001$, ****$p < 0.0001$

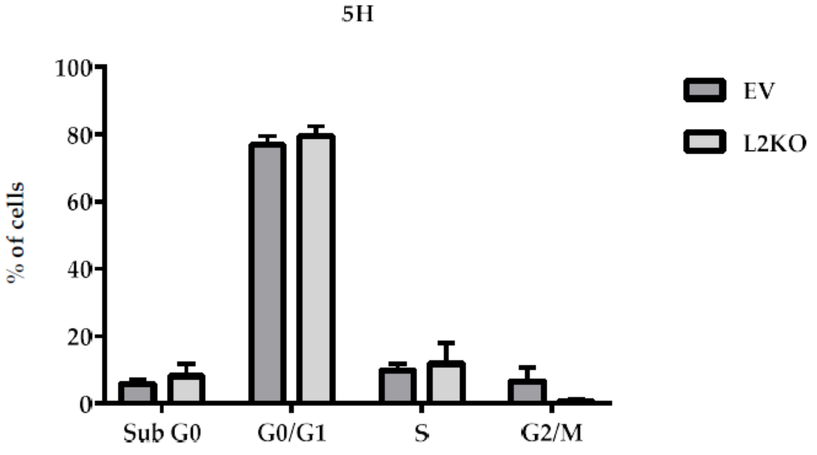

Figure S6. Cell cycle analysis of L2KO vs. EV cells to verify the efficiency of 3 days of TSH deprivation. Cells were starved as detailed in M&M. The data show that about 90% of L2KO and EV cells were in the G0/G1 phase, as expected.

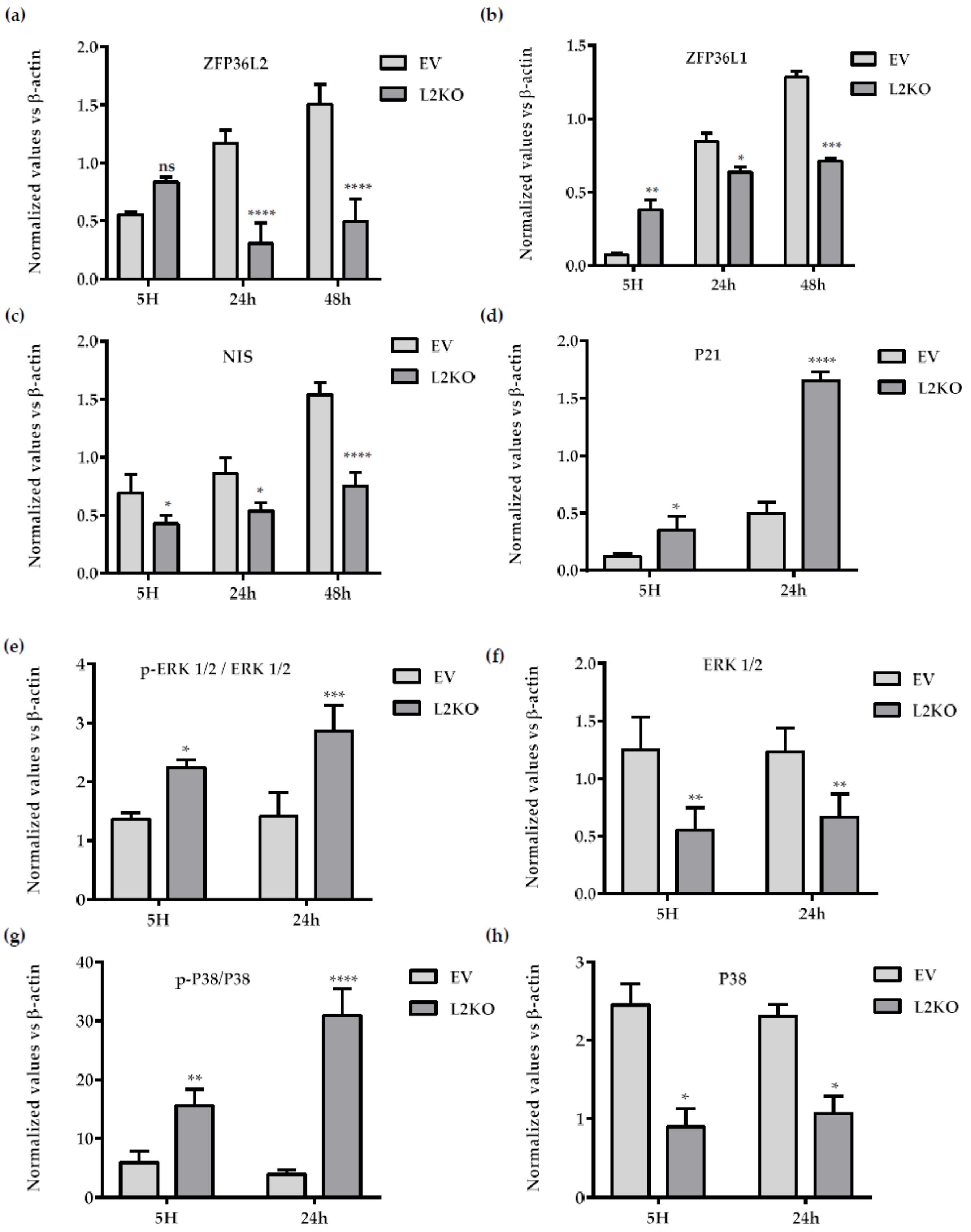

Figure S7. (**a–c**) Densitometric analysis of Western blotting experiments shown in Figure 7a. *$p < 0.05$; **$p < 0.005$; *** $p < 0.001$; ****$p < 0.0001$; ns is for not significant. (**b–h**) Densitometric analysis of Western blotting experiments shown in Figure 7e,f. * $p < 0.05$; ** $p < 0.005$; *** $p < 0.001$; **** $p < 0.0001$.

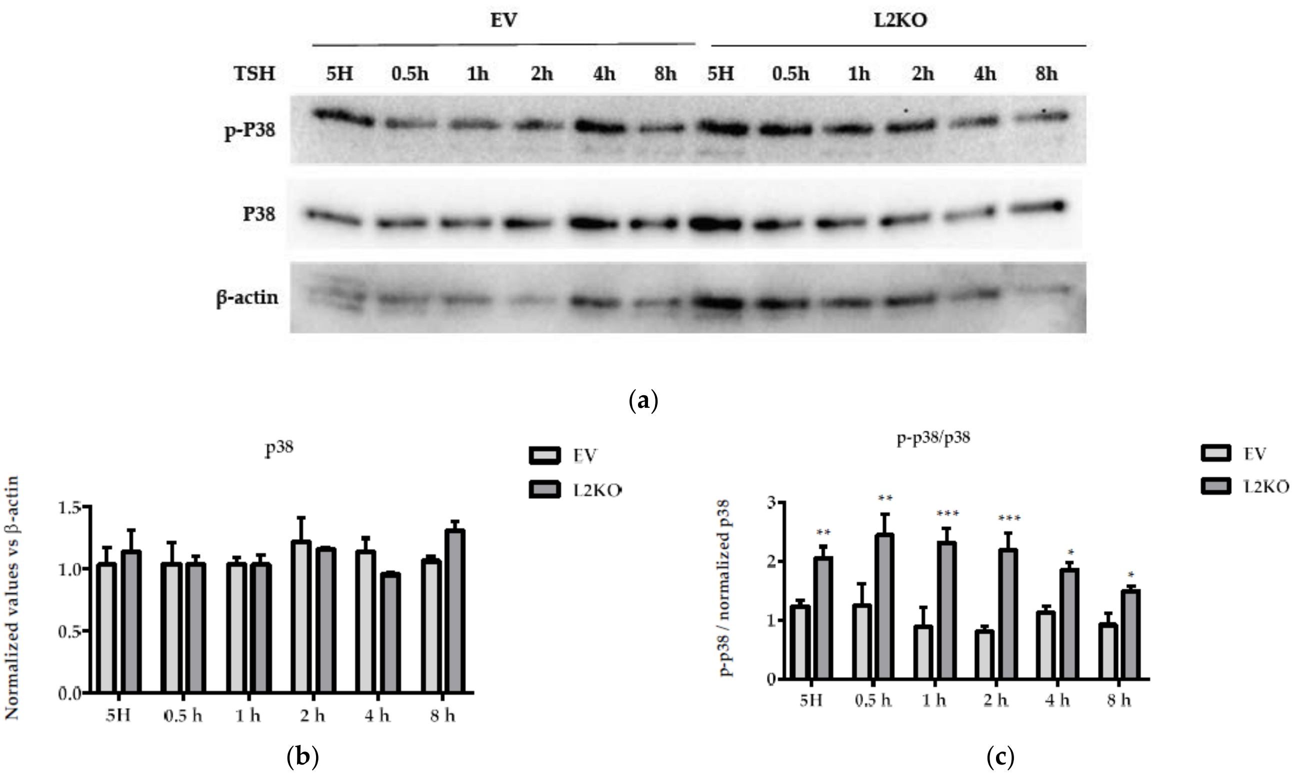

Figure S8. Analysis by Western blotting of the activation of P38-MAPK pathways at early times of TSH stimulation. EV and L2KO were starved and stimulated with TSH as described in M&M. At the reported time points the cells were collected, lysed in RIPA buffer, and analyzed by Western blotting (**a**). Densitometric analysis of total P38 protein of figure S8a (**b**) and of the p-P38 vs total P38 ratio of the same figure (**c**). *$p < 0.05$; **$p < 0.005$; ***$p<0.001$.

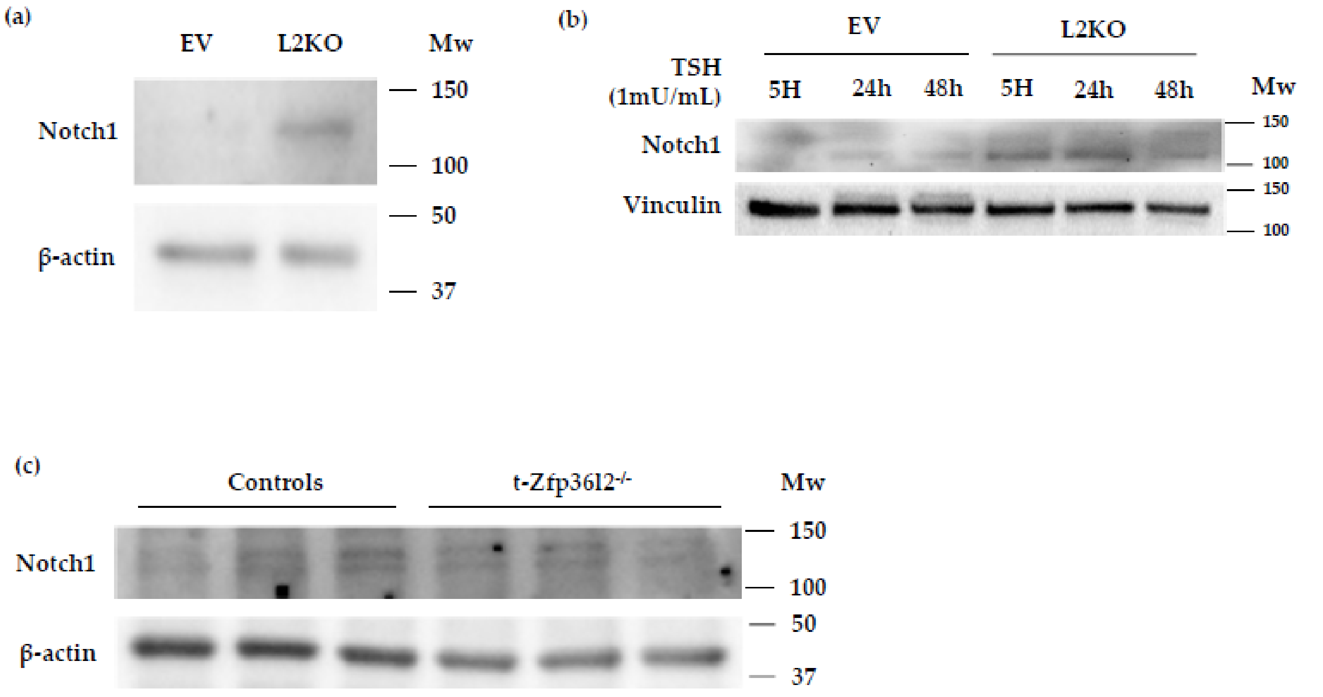

Figure S9. (**a**) Western blotting analysis of cleaved-Notch1 in experimental points shown in Figure S8a. (**b**) Western blotting analysis of cleaved-Notch1 in experimental points shown in Figure S8b. (**c**) Western blotting analysis of cleaved-Notch1 in experimental points shown in Figure S8c.

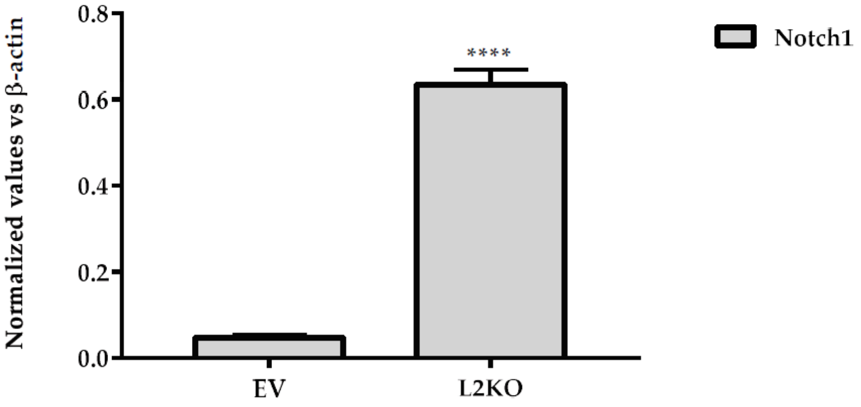

(**a**)

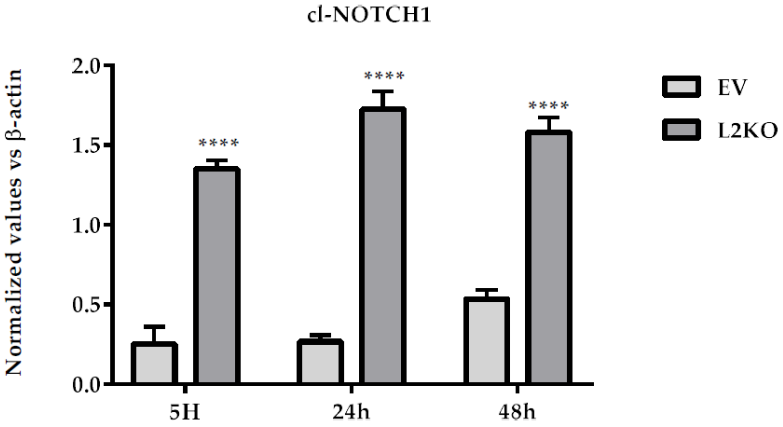

(**b**)

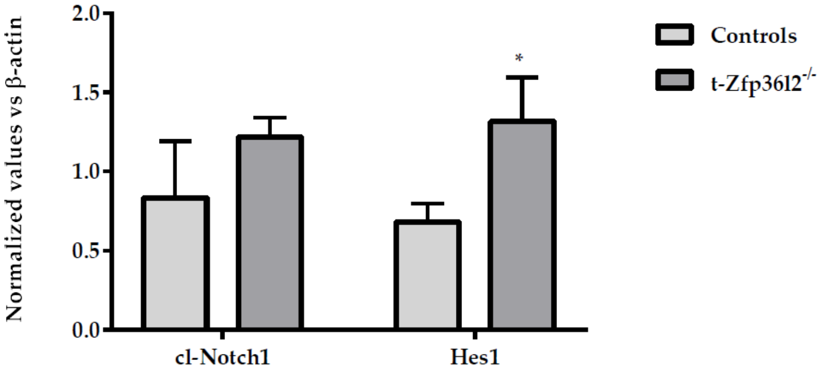

(**c**)

Figure S10. (**a**) Densitometric analysis of western blotting experiments showed in Figure S9a. (**b**) Densitometric analysis of western blotting experiments showed in Figure S9b. (**c**) Densitometric analysis of western blotting experiments showed in Figure S9c (for cl-Notch1) and Figure 8d (for Hes1). *$p < 0.05$; ****$p<0.0001$.
